# The impact of a community-based risky drinking intervention (Beat da Binge) on Indigenous young people

**DOI:** 10.1186/s12889-015-2675-4

**Published:** 2015-12-30

**Authors:** Thameemul Ansari Jainullabudeen, Ailsa Lively, Michele Singleton, Anthony Shakeshaft, Komla Tsey, Janya McCalman, Christopher Doran, Susan Jacups

**Affiliations:** National Drug and Alcohol Research Centre, UNSW Australia, Sydney, NSW 2052 Australia; Gindaja Treatment Centre, Yarrabah, Qld Australia; The Cairns Institute, James Cook University, Townsville City, QLD 4811 Australia; School of Human Health and Social Sciences, Central Queensland University, 160 Ann Street Brisbane, QLD 4000 Brisbane, Australia

**Keywords:** Indigenous population, Alcohol, Binge drinking, Substance misuse, Intervention studies

## Abstract

**Background:**

Alcohol misuse imposes substantial harm on Indigenous Australians whose health status is poorer than non-Indigenous Australians. Although Indigenous youth are over represented in Indigenous alcohol harms, few interventions addressing alcohol-related harm among Indigenous youth have been evaluated. Given this paucity of evidence, a survey was designed to evaluate the effects of a whole-of-community, anti-binge drinking intervention for young people in an Indigenous community in far north Queensland, Australia.

**Methods:**

A cross sectional, baseline-post intervention study assessed the impact of a two year anti-binge drinking intervention targeting young people (18–24 years). A survey was developed and implemented at baseline and again two-years post-intervention, administered by young local people employed as research assistants. Survey respondents were recruited through snowballing techniques. Survey items asked about respondents’ knowledge of binge drinking and standard drinks, involvement in alcohol-free social activities, frequency of short-term risky drinking (binge drinking), and mean alcohol expenditure during short-term risky drinking occasions.

The intervention was called Beat da Binge. Two major events and multiple minor activities each year were implemented, focusing on drinking education, alcohol-free community-wide social events, and youth-specific sporting and social activities to facilitate self-empowerment.

**Results:**

Beat da Binge was associated with a statistically significant 10 % reduction in the proportion of survey respondents who reported that they had engaged in an episode of short-term risky drinking, in the frequency of short-term risky drinking for all beverage types except wine (ranging from 4 % to 31 % reductions), in mean expenditure on alcohol during short-term risky drinking sessions ($6.25) and in the proportion of activities with family/friends that usually include alcohol (7 %). There were also statistically significant increases in awareness of binge drinking and standard drinks (28 % and 21 % respectively). In addition to alcohol-specific outcomes, there was a statistically significant 8 % increase in the proportions of respondents engaged in training as their main weekday activity, which was partly off-set by a 13 % reduction in those whose main weekday activity was family care or home-related tasks.

**Conclusions:**

Reductions in the proportion of survey respondents who reported binge drinking, along with increases in awareness and involvement in alcohol-free social activities suggest the community-based intervention was effective. The potential impact of sample selection and self-reporting limitations on results need further investigation. There is an urgent need for Indigenous, community-driven public health programs that are well evaluated to both improve Indigenous health and the strength of the current evidence base to inform future community interventions.

## Background

Among Indigenous Australians, short-term risky drinking, currently defined by the National Health and Medical Research Council (NHMRC) of Australia as the consumption of more than four standard drinks in a single drinking occasion [1], appears to be a particularly problematic pattern of drinking: 25 % of Indigenous Australians aged at least 14 years reported drinking at short-term risky drinking at least once a week prior to a 2010 national survey, which was higher than the rate (16 %) reported for non-Indigenous Australians [2]. A disproportionately high burden of harm associated with short-term risky drinking is borne by young Indigenous Australians (aged 15–34 years), where alcohol is associated with the greatest burden of disease and injury for males, and the second greatest burden of disease and injury for females [3]. Indigenous youth are more than twice as likely as their non-Indigenous counterparts to die from alcohol-attributable causes [4, 5] and alcohol-related social problems are disproportionately higher among Indigenous, compared to non-Indigenous, youth [6].

Despite the recognised extent of these harms, the specific alcohol consumption and harm characteristics of Indigenous young people living in a diverse range of communities have not been adequately described [7]. In general evaluations in peer-reviewed journals of young Australian Indigenous substance misuse interventions is also lacking [8]. Consequently, the tailoring and evaluation of interventions aimed at modifying harmful drinking behaviours among young people in different Indigenous communities could be more precise, if improved and more standardised measures of risky drinking and alcohol harms were developed [9].

In 2008, the Australian Government provided funding through a National Binge Drinking Strategy for programs designed to reduce rates of short-term risky drinking among Indigenous young people. Yarrabah, an Indigenous community approximately a one-hour drive from Cairns in Queensland, successfully applied for funding to develop and implement a community-based intervention targeting short-term risky binge drinking among their young people. Key stakeholders in Yarrabah were concerned about the apparent increase in risky alcohol consumption among their young people following the cessation of funding for the Australian Government’s Community Development Employment Program (CDEP). The lead organisation for this intervention in Yarrabah (Gindaja Treatment Centre) invited researchers from James Cook University (JCU) in Cairns, the National Drug and Alcohol Research Centre (NDARC) at the University of New South Wales (UNSW) in Sydney, and the Hunter Medical Research Institute (HMRI) at the University of Newcastle, to partner with them to evaluate their program.

This study aims to evaluate the effects of a whole-of-community, anti-binge drinking intervention for young people in Yarrabah.

## Methods

### Ethics

The protocol for the research project was approved by JCU Human Research Ethics Committee (approval number H 3532).

### Setting

In 2011, Yarrabah had a population of 2,409: 97 % were Indigenous and half the population was aged 25 years or less, with a median age of 21 years [10]. According to the Australian Socio-Economic Indexes for Areas (SEIFA), a composite index based on average rates of income, job status, occupation, personal qualifications, service availability and housing conditions, Yarrabah was Australia’s most disadvantaged local government area in 2011 [11].

### Study design

Given the uniqueness of the intervention to Yarrabah and for practical limitations, largely funding constraints, a baseline/post evaluation design was agreed using self-reported outcomes.

### Intervention

Beat da Binge, a two-year project targeting binge drinking, commenced in April 2010. Key features of the intervention are that it was community-driven, utilised participatory approaches, actively engaged young people in its design, implementation and evaluation, and sought to create a partnership with researchers [12]. Intervention activities covered three broad themes: raising awareness of safe drinking practices; promotion of enjoyable alcohol-free activities as alternatives to alcohol inclusive events; and diversionary activities to alleviate boredom and motivate achievement and self-empowerment [12]. Beat da Binge activities were implemented to coincide with two major events and at least twelve minor events each year.

Major events included Foundation Day (the commemoration of the founding of the mission in 1893) and NAIDOC week (National Aborigines and Islanders Day Observance Committee which celebrates the history, culture and achievements of Aboriginal and Torres Strait Islander peoples). Activities coinciding with major events were the distribution of T-shirts, leaflets and other resources that specifically conveyed messages about safer drinking practices, high-risk times for risky drinking and harms, and provided ideas for alternatives to drinking. Project staff was also available to actively promote and discuss these messages. Similar activities were implemented as minor community events in two ways, first, by integrating them into existing events, such as domestic violence week, drug action week, child protection week, carols by candlelight, rugby league carnival day, Christmas movie night, the Bishop Malcolm Indigenous carnival, and the healthy community initiative of community survival day. Second, a range of events were offered with co-sponsorship from local organisations, including music, sporting and cultural events, a ‘dive-in’ movie night, Seahawks chickettes activity, self-defence training and restraint techniques (START), taekwondo, a touch-football camp, and boxing.

### Measures

Baseline and post-intervention surveys were developed to target 18–24 year olds in Yarrabah. This age group was chosen to coincide with the minimum legal drinking age (lower end of the range) and because approximately half the population of Yarrabah is aged less than 25 years (upper end of the range). The Beat da Binge project officers, who were local residents, trained as research assistants, consulted with people in this age range in Yarrabah to identify potential survey items and potential ways of phrasing those items. In partnership with researchers, those potential items were then aligned with survey items that have some evidence for their reliability, validity or comparability with existing surveys. Finally, they were organised into broad domains, including demographics (summarised in Table 1), and alcohol consumption and related behaviours (summarised in Tables 2 and 3).

**Table 1:** Demographic and alcohol use characteristics of 18–24 year old Indigenous residents in an Indigenous community in Far North Queensland at baseline (*N*=218) and post- (*N*=154) intervention

Characteristic		Baseline survey	Post-intervention survey	Difference	Odds Ratio (Confidence Interval)	Chi-square/*t*-test statistic	*p*-value
		*n* (%)	*n* (%)	%			
Gender^b^	Male (%)	107 (49 %)	76 (49 %)	0.0	0.99 (0.66–1.58)	0.13	0.94
	Female	109 (50 %)	76 (49 %)	−0.6			
Age (years)	Mean (SD)	21.2 (2.1)	21.3 (2.3)	0.1		1.09	0.27
Number in household	Mean (SD)	7.2 (2.9)	6.9 (3.5)	−0.3		1.13	0.26
Income (AUS$)	Mean (SD) income per fortnight	$498.37 ($467.16)	$529.45 ($503.92)	$31.08		−0.56	0.57
		(*n*=186)	(*n*=126)				
	Main source of income^a,c^:						
	*Paid work*	19 (8.7 %)	23 (15 %)	6.2	0.56 (0.27 – 1.12)	3.40	0.18
	*Social security benefits*	180 (83 %)	121 (79 %)	−4			
Main weekday activity	Family care or home related	110 (51 %)	58 (38 %)	−12.8	0.63 (0.40–0.98)	4.58	**0.03**
	Job (Full time/Part time/casual)	26 (12 %)	22 (14 %)	2.4	1.23 (0.64 – 2.36)	0.45	0.50
	Training	13 (6 %)	21 (14 %)	7.7	2.48 (1.14 – 5.57)	6.33	**0.01**
Education	Tertiary training/qualifications	42 (19 %)	36 (23 %)	4.1	1.55 (0.90 – 2.66)	2.82	0.09
Alcohol consumption status^d^	Drinks alcohol	172 (79 %)	124 (81 %)	1.6	1.11 (0.64 – 1.92)	0.15	0.70
	Short-term risky drinker	152 (70 %)	92 (60 %)	−10	0.64 (0.41 – 0.99)	3.99	**0.05**
	(% of total)						
	Abstainer	40 (18 %)	25 (16 %)	−2.1	0.86 (0.48 – 1.54)	0.28	0.60

^a^Respondents may have provided multiple responses and therefore number and proportion may not add up to total

^b^2 respondents (baseline) and 2 respondents (post-intervention) did not answer this question

^c^15 respondents (baseline) and 9 (post intervention) did not answer this question

^d^6 respondents (baseline) and 5 respondents (post intervention) did not answer this question

**BOLD** – statistically significant at 95 % level

**Table 2:** Frequency of short-term risky drinking^a^ by beverage type, at baseline (*N*=152) and post- (*N*=92) intervention

Beverage type	Level of short-term risky drinking	Frequency of drinking sessions at as such risky levels	Baseline sample	Post intervention sample	Difference	Odds Ratio (Confidence Interval)	Chi-square/*t*-test statistic	*p*-value
			*n* (%)	*n* (%)	%			
Beer	At least 4 full strength bottles/cans (375ml) in single drinking session	never^b^	3 (2.0 %)	7 (8.7 %)	5.6	0.18 (0.04 – 0.74)	7.06	**0.03**
		monthly/less than monthly	45 (30 %)	17 (19 %)	−11			
		at least weekly	33 (22 %)	16 (17 %)	−4.3			
UDL/mixed drinks	At least 4 bottles/cans (375ml) in single drinking session	never^b^	1 (0.7 %)	5 (5.4 %)	4.8	0.07 (0.00 – 0.60)	18.6	**<0.01**
		monthly/less than monthly	81 (54 %)	40 (44 %)	−10			
		at least weekly	64 (42 %)	10 (11 %)	−31			
Spirits	At least one quarter of a bottle (750ml) in single drinking session	never^b^	2 (1.3 %)	4 (4.3 %)	3.0	0.17 (0.03–0.95)	6.73	**0.03**
		monthly/less than monthly	86 (57 %)	34 (37 %)	−20			
		at least weekly	53 (35 %)	13 (14 %)	−20			
Wine	At least 4 glasses (125ml) in single drinking session	never^b^	3 (2.0 %)	3 (3.3 %)	1.3	0.73 (0.13–4.08)	2.99	0.22
		monthly/less than monthly	9 (5.9 %)	11 (12 %)	6.0			
		at least weekly	13 (8.6 %)	5 (5.4 %)	−3.1			

^a^More than 4 standard drinks per single occasion of drinking

^b^reference category

**BOLD** – statistically significant at 95 % level

**Table 3:** Alcohol consumption characteristics of short-term risky drinkers^a^, at baseline (*N*=152) and post-intervention (*N*=92)

Characteristic	Baseline sample	Post intervention sample	% or $ difference	Odds Ratio (Confidence Interval)	Chi-square/*t*-test statistic	*p*-value
	*n* (%)	*n* (%)				
Mean expenditure on alcohol during binge drinking session (AUS$) (SD)	$98.69 ($39.51)	$92.44 ($93.08)	$6.25		−2.96	**<0.01**
Binge drinking awareness	39 (26 %)	48 (54 %)	28	3.83 (2.09–7.05)	22.4	**<0.01**
Familiarity with standard drinks measure	63 (41 %)	54 (62 %)	21	2.54 (1.41 – 4.62)	18.3	**<0.01**
Activities with family/friends include alcohol	85 (56 %)	32 (36 %)	−7	0.45 (0.25 – 0.80)	8.53	**<0.01**

^a^More than 4 standard drinks per single occasion of drinking

**BOLD** – statistically significant at 95 % level

Items for alcohol consumption, for example, were measured using the three-item Alcohol Use Disorders Identification Test (AUDIT-C) [13], which was tailored to the literacy levels and social norms for Yarrabah young people. To identify quantity of alcohol consumption per occasion (AUDIT-C question two), respondents were first asked to identify their beverage preference (beer, UDL/mixed drinks, mixed spirits, or wine). For each preferred beverage type, they were then asked to identify the strength of the alcohol they usually consume (full, mid or low); the type of container from which they typically drink (e.g. 375ml cans/stubbies for beer and UDL/mixed spirits); the amount of alcohol beverage they would normally pour into a 425ml glass for spirits and wine (by drawing a line on a picture of a glass); the number of those glasses they would normally consume in a session; and the volume of spirits remaining in a 750ml bottle after a drinking session (by drawing a line on a picture of a bottle). Respondents were also asked about the frequency with which they consume alcohol (AUDIT-C question one) and the frequency with which they consume more than four standard drinks in a session (AUDIT-C question three, using the cut-off of four standard drinks to align with the Australian NHMRC drinking guidelines).

Items for behaviours directly related to alcohol consumption included the amount of money normally spent on alcohol in a drinking session and the proportion of activities with family and friends that would normally include drinking alcohol.

### Procedure

The details of the procedure are provided elsewhere [12]. In summary, young people in Yarrabah were trained in interview techniques and remunerated as research assistants to opportunistically survey other young people (18–24 years) at Beat da Binge events, in the park, and with friends and family members. Four were employed to conduct, baseline surveys (March 2011) and seven post-intervention surveys (May 2012). Survey respondents were recruited through snowballing techniques. Participants received an information sheet about the research and all signed consent forms prior to being asked to respond to the survey items. Since responses were not identifiable, the baseline and post-intervention surveys represent two cross-sectional surveys.

### Statistical analysis

Data collected from both the baseline and post-intervention surveys were summarised and differences reported. Differences in the proportion of respondents from baseline to post-intervention are reported for: involvement in at least one session of short-term risky drinking in the previous 12 months; involvement in at least one session of short-term risky drinking in the previous 12 months by beverage type; and, for those who had engaged in at least one session of short-term risky drinking in the previous 12 months, mean expenditure on alcohol during short-term risky drinking sessions, awareness of what binge drinking is, familiarity with standard drinks and the proportion of activities with family or friends that include alcohol. Finally, as a measure of exposure to the intervention, the proportion of post-intervention respondents who indicated they had heard of Beat da Binge is reported. Secondary outcomes include changes in: mean expenditure on alcohol during binge drinking sessions; binge drinking awareness; familiarity with standard drinks; and the extent to which activities with family and friends included alcohol. Inferential statistical analysis were conducted using Chi-square (*χ*^2^) tests, Student’s *t*-test test and Odds Ratios (OR). Statistical significance was set as *p*<0.05 (95 % level of significance). All analyses were conducted using STATA [14].

## Results

### Exposure to Beat da Binge

Beat da Binge events attracted an estimated 1,880 participants and 78 % of respondents to the post-intervention survey reported that they had heard of the project.

### Demographic and alcohol use characteristics of the total samples

Table 1 shows participants in the baseline (*n*=218) and post- (*n*=154) intervention surveys reported similar demographic characteristics. Respondents for the post-intervention survey were statistically significantly less likely to be engaged in family care or home-related tasks as their main weekday activity (OR=0.63, 95 % CI: 0.40-0.98, *p*=0.03) and more likely to be engaged in training (OR=2.48, 95 % CI: 1.14-5.57, *p* =0.01). For alcohol use, there was a statistically significant 10 % reduction in the proportion of respondents who reported engaging in short-term risky drinking after the implementation of the Beat da Binge program, relative to the baseline proportion (OR=0.64, 95 % CI: 0.41–0.99, *p*=0.05).

### Frequency of short-term risky drinking by beverage type

For those who reported short-term risky drinking, Table 2 shows statistically significant reductions from baseline (*n*=152) to post (*n*=92) implementation of the Beat da Binge program in the proportion of respondents who engaged in short-term risky drinking, across all beverage types except wine: beer (30 % and 22 % in baseline to 19 % and 17 % in post intervention, refer to Table 2 for detailed statistical significance); UDL/mixed drinks (81 % and 64 % in baseline to 40 % and 10 % in post intervention); and spirits (57 % and 35 % in baseline to 37 % and 14 % in post intervention). Reductions were greatest for UDL/mixed spirits and spirits, which were the two most popular alcoholic beverages among respondents.

### Alcohol consumption characteristics of short-term risky drinkers

For those who reported short-term risky drinking, Table 3 shows statistically significant reductions from baseline- to post-intervention in mean expenditure on alcohol in a drinking session (*t*= −2.96, *p*<0.01) and the proportion of respondents who reported that their activities with family/friends usually include alcohol (56 % in baseline to 36 % in post intervention, refer to Table 3 for detailed statistical significance). There were statistically significant increases in the proportion of respondents who reported that they had heard about binge drinking (26 % in baseline to 54 % in post intervention) and who were able to identify a standard drink (41 % in baseline to 62 % in post intervention).

## Discussion

### Key findings

The implementation of the Beat da Binge program in Yarrabah coincided with a statistically significant 10 % reduction in the proportion of survey respondents who reported that they had engaged in an episode of short-term risky drinking, in the frequency of short-term risky drinking for all beverage types except wine (ranging from 4 % to 31 % reductions), in mean expenditure on alcohol during short-term risky drinking sessions ($6.25) and in the proportion of activities with family/friends that usually include alcohol (7 %). There were also statistically significant increases in awareness of binge drinking and standard drinks (28 % and 21 % respectively). In addition to alcohol-specific outcomes, there was a statistically significant 8 % increase in the proportions of respondents engaged in training as their main weekday activity, which was partly off-set by a 13 % reduction in those whose main weekday activity was family care or home-related tasks.

### Limitations

The baseline/post evaluation design was the most pragmatic option but its primary limitation is that it cannot rule out the possibility that a factor other than the Beat da Binge program was causally related to the observed outcomes, even though there was no other intervention implemented in Yarrabah during the project period. The challenges of using a systematic or random sampling in an outer regional Indigenous community forced the study to use convenience sampling approach and similar challenges have been faced in a remote Indigenous study on substance use [15]. Nevertheless, 59 % of 18–24 youths in the community participated in the baseline survey. Potential impact of sample selection or self-reporting limitations on the results could not be verified using relevant existing community level data (e.g. participation in training) since it was lacking. The outcomes rely on self-reported survey data which are highly vulnerable to selection, response and social desirability biases and seasonal differences. However the survey items were aligned as closely as possible with self-report measures that do have demonstrated reliability and validity, even for Indigenous Australians, such as AUDIT-C [16].

### Implications

While national data are available on the extent and nature of the Indigenous youth binge drinking problem, there is a dearth of community specific data that would allow programs to be targeted to local risk factors and harms, and to evaluate community-level interventions [8]. Despite the substantial limitations of this study, it does represent one of the first attempts to utilise a community designed and implemented survey instrument to evaluate the impacts of a community designed and implemented intervention. It has demonstrated the feasibility of such an approach and highlights a model of Indigenous led intervention research that partners, at the request of an Indigenous community itself, with researchers who have specific expertise in utilising evaluation designs and appropriate and practical measures [17]. It highlights the potential to replicate this approach in multiple communities in a co-ordinated way that would allow the use of evaluation designs that provide greater experimental control than basline/post evaluations, such as multiple baseline designs [18] or RCTs [16].

Community stakeholders do not dispute the findings of this study and indicated, during presentation of study results, that the reported changes are possibly true and that they have observed positive changes amongst youths. Beyond the potential benefits of reduced proportions of short-term risky drinkers among young people in Yarrabah, this study suggests that projects led and designed by Indigenous communities, and evaluated in partnership with researchers, can be potentially effective. This integrated approach may provide a more effective blue-print for reducing alcohol-related harm than the traditional government- or researcher-designed policies and programs that typically allow insufficient community input. Local leaders in Yarrabah consider that this community-driven approach, with active engagement of young people in its design, implementation and evaluation, is a key reason for the positive outcomes observed. Such locally-developed interventions and evaluations not only provide appropriate education, in this case regarding binge drinking, standard drinks and alcohol-related harms, but may also contribute to improving the social determinants of health by providing training and employment to young people as casual researchers and increasing their locus of control. There is international evidence that the empowering nature of such initiatives can, in itself, lead to improved health outcomes, and that empowerment is a viable public health strategy [19].

## Conclusions

This study reports on the collection and use of community specific data to assess the impact of a community designed and implemented intervention for Indigenous young people engaging in short-term risky drinking. The primary outcome of a statistically significant 10 % reduction in the proportion of Indigenous young people who reported that they had engaged in an episode of short-term risky drinking suggests that locally developed interventions can be potentially effective, and that encouraging partnerships between Indigenous communities and researchers to evaluate community-led intervention is feasible. Furthermore, statistically significant secondary outcomes included increased awareness of binge drinking and standard drinks, and greater engagement in social activities that did not involve alcohol consumption. The potential impact of sample selection and self-reporting limitations on results need further investigation. This study provides further evidence of the feasibility and potential effectiveness of Indigenous-led, community-based intervention and evaluation.
